# “I Know What I Need”: A Mixed Methods Study of Mental Health-Seeking Behaviors in Formerly Incarcerated Black Men

**DOI:** 10.1007/s40615-025-02591-7

**Published:** 2025-08-13

**Authors:** Helena A. Addison, Therese S. Richmond, Toorjo Ghose, Sara F. Jacoby

**Affiliations:** 1 School of Nursing, University of Pennsylvania, Philadelphia, PA, USA; 2 National Clinicians Scholars Program, Yale University, New Haven, CT, USA; 3 School of Social Policy and Practice, University of Pennsylvania, Pennsylvania, PA, USA

**Keywords:** Incarceration, Mental health, Black men, Health disparities, Mixed methods

## Abstract

**Background:**

Black men in the United States experience disproportionate rates of incarceration, contributing to adverse mental health outcomes. Yet, research on mental health-seeking behaviors of formerly incarcerated Black men (FIBM) remains limited, often focusing on formal mental health services, leaving knowledge gaps about coping strategies, support networks, and the influence of incarceration experiences on health-seeking behaviors.

**Purpose:**

The purpose was to describe how incarceration shapes FIBM’s health-seeking behaviors for mental health needs.

**Methods:**

This convergent mixed methods study collected and analyzed data from semi-structured interviews and surveys with 29 FIBM. Reflexive thematic analysis and descriptive statistics were used; integrated data analysis examined the relationship between qualitative findings and quantitative measures.

**Results:**

Five themes described how incarceration shaped health-seeking behaviors for FIBM: (1) “I know what I need” reflected self-assessment of mental health needs; (2) “You learn to depend on yourself” described self-reliance and coping strategies; (3) “Trying to express myself” reflected efforts to engage social support; (4) “How can you put your trust in that?” highlighted mistrust in carceral mental health services due to historical and personal experiences of harm; and (5) “It’s not normal for guys like us” described how participants’ navigate stigma and additional barriers in seeking community-based mental healthcare. Integrated data analysis revealed a nuanced understanding of how FIBM expressed their mental health needs compared to their scores on mental health measures.

**Conclusion:**

This study shows how FIBM identify and address mental health needs across individual, social, and structural contexts, providing insights for improving mental health outcomes.

## Introduction

Over 6 million Black men in the United States (U.S.) have been incarcerated at some point in their life [1] and often have worse mental health outcomes than Black men who have never been incarcerated [2]. Among Black men, incarceration history is associated with higher levels of psychological distress, increased severity of depressive and posttraumatic stress disorder (PTSD) symptoms, and delayed mental health treatment [2–8]. Connecting formerly incarcerated Black men (FIBM) to strategies and interventions that address their mental health needs following incarceration could be a crucial pathway to supporting their overall health and well-being. However, there are critical limitations in current understandings of how to identify and address mental health needs in FIBM.

Formerly incarcerated individuals with documented mental illness face a heightened risk of negative health outcomes, including a higher likelihood of emergency department visits for mental health crises and a greater risk of substance overdose-related deaths [9, 10]. Additionally, reincarceration rates are significantly higher among individuals who experience mental illness post-release [11, 12]. To address these downstream consequences of poor mental health in FIBM, it is essential to understand their health-seeking behaviors, or how they identify, make decisions, and act on their mental health needs to reduce disease burden, prevent health complications, and improve well-being [13, 14].

Health-seeking is an interactional process, informed by social, cultural, and structural context [13, 14]. Research examining mental health of FIBM has primarily relied on standardized mental health measures to identify mental health needs [2]. However, such measures have been criticized for underestimating or misclassifying the mental health needs of Black individuals because they fail to account for cultural norms, the influence of anti-Black racism, and other contextual factors [15–19]. Qualitative studies have begun to illuminate sociocultural norms and structural factors, including incarceration, that may influence FIBM’s presentation or identification of mental health symptoms, and how they define mental health overall [17, 18, 20–22].

Even when mental health needs are identified, they often persist due to limited access to care. The relative underuse of formal mental health services among Black men is due to a variety of factors, ranging from mental health stigma to time and financial constraints [23–27]. Unidentified and unaddressed mental health needs in FIBM have also been linked to the larger historical and contemporary structural context of mental health services in the U.S., including anti-Black racism in the classification of and disproportionate criminalization of mental illness experienced by Black people [15, 28]. Research has also identified ways that implicit biases and lack of cultural humility during the provision of mental health services reifies barriers to mental health care for Black men in the U.S. [15, 29, 30].

Despite the prevalence of incarceration history among Black men and associated mental health risks, the role of incarceration in health-seeking is rarely examined [2, 25]. A period of incarceration could theoretically act as a barrier or facilitator of health-seeking for mental health needs after incarceration. Although incarcerated people are constitutionally guaranteed healthcare including mental healthcare, access to mental health services during incarceration is often inconsistent and experiences may be perceived as inadequate, inappropriate, or punitive [31–33]. These experiences may deepen mistrust in formal mental health services, especially in the context of competing priorities of safety, housing, and employment after incarceration [25, 34–36].

In order to understand the scope of health-seeking for mental health needs, it is imperative to think beyond uptake of formal mental health services to include individual coping behaviors or support from family and friends. Research with Black men has shown that they often rely on informal sources of support including from family and religious leaders [37]. However, FIBM specifically report experiences of social isolation and having fewer sources of social support after incarceration [21, 38, 39].

This study describes how incarceration history shapes health-seeking intentions and behaviors by understanding how FIBM identify their mental health needs and use their resources to address these needs. Efforts to eliminate health disparities experienced by FIBM, require in-depth and comprehensive understanding of health-seeking behaviors and the impact of incarceration as a driver of racial health inequities.

## Methods

### Study Design

This convergent mixed method (QUAL + quan) research design integrates in-depth semi-structured interviews and embedded structured surveys to comprehensively examine the complex factors that influence health-seeking behaviors among FIBM [40–42]. This study was approved by the University Of Pennsylvania Institutional Review Board.

### Participants

Study inclusion criteria were (1) self-identified as a Black adult man, (2) lived in Philadelphia, PA, and (3) had a history of incarceration.

### Recruitment

Participants were recruited through convenience and snowball sampling strategies. A research assistant who was a FIBM and a certified peer specialist trained in recovery and trauma-informed care provided potential participants information about the study. Interested individuals contacted the principal investigator, who provided details, answered questions, and scheduled virtual interviews. Additionally, two reentry organizations shared study information with their clients. Study participants were encouraged to share information about the study with their network—to enable recruitment for snowball sampling [43]. In total, 24 participants were recruited through the research assistant or reentry organizations and 5 were recruited through snowball sampling. The sample size of 29 was justified using the concept of information power, which guides sample size decisions, as the qualitative data offered sufficient richness and depth to address the study aims [44, 45]. Informed consent was obtained from all participants included in the study.

### Data Collection

Each participant participated in one interview. One-on-one interviews included open-ended qualitative questions and close-ended survey questions, lasted approximately 1 hour, and were audio recorded; survey data were recorded using Qualtrics. Participants received a $50 gift card and information about local, free or low-cost behavioral health resources for their participation in this study.

The semi-structured interview guide included 12 open-ended qualitative questions or prompts that addressed participants’ perceptions and experiences of mental health, health-seeking behaviors, and engagement in mental healthcare. These questions were derived from key constructs from previous research on mental health, health-seeking behaviors, and engagement in mental healthcare, and further informed by the clinical and research expertise of the research team. To support the rigor of the interview content, the interview guide was reviewed and refined in pilot implementation with a member of the research team who is a FIBM. Example interview questions and prompts included, “tell me about how you handle difficult situations or circumstances” and “how do you deal with any negative feelings or emotions that arise from those situations?”

The interview also included closed-ended survey questions that captured sociodemographic characteristics (e.g., age), incarceration history (e.g., total duration of incarceration), and mental health treatment history along with measures of their current mental health and health-related quality of life (HRQoL). Depression was assessed using the Patient Health Questionnaire 2 (PHQ-2), a 2-item rating scale that measures the severity of depressive symptoms [46, 47]. Scores range from 0 to 6 with higher scores indicating higher symptom severity [46]. Anxiety was assessed using Generalized Anxiety Disorder 2-item Questionnaire (GAD-2), which measures the severity of anxiety symptoms with scores ranging from 0 to 6 with higher scores indicating greater symptom severity [47, 48]. For the PHQ-2 and GAD-2, scores of 3 or higher indicated the need for further screening. The Primary Care PTSD Screen for DSM-5, (PC-PTSD-5) is a 5-item self-report rating screen to help identify individuals with probable PTSD [49]. Scores range from 0 to 5 with higher scores indicating higher symptom severity, and a score of 3 or more indicating probable PTSD [49]. The Medical Outcomes Study: 8-item Short Form Survey Instrument (SF-8) is an 8-item rating scale used to measure quality of life in 8 domains (general health, physical health, mental health, bodily pain, vitality, and physical, social, and role functioning) [50]. Norm-based scores range from 0 to 100 with higher scores indicating better HRQoL [50]. This study focused on the mental component score (MCS-8) in comparison to population norms. The survey also assessed experiences postponing or forgoing needed mental or physical healthcare, any instances of racial or criminal record discrimination in healthcare settings, and mental health treatment history. Survey questions were administered by the interviewer following the open-ended portion of the interview to ensure complete responses to each item.

### Data Analysis

Interview recordings were transcribed, checked for accuracy and then analyzed using NVivo software [51]. Qualitative data were analyzed using thematic analysis procedures outlined by Braun and Clark, including generating initial codes, categorizing codes to develop themes, and reviewing and refining themes to adequately reflect data [44, 52]. Three researchers contributed to data analysis. The first author conducted initial coding of all interviews in collaboration with the senior author, a qualitative research expert. A second coder independently analyzed six interviews (20% of the dataset) to refine codes and establish intercoder reliability [53, 54]. Themes were developed iteratively and reviewed with mentors and peers for credibility and rigor was maintained using Braun and Clarke’s evaluation criteria for thematic analysis [44]. Example quotes included in this manuscript were edited for clarity and brevity. Quotes are attributed to pseudonyms selected by participants, with any duplicates or real names modified to ensure differentiation and confidentiality.

Quantitative survey data were collected in Qualtrics [55] and exported to Stata [56] for descriptive analysis. Summary statistics, including means, standard deviations, medians, and ranges were calculated for continuous variables. Frequencies and proportions/percentage were calculated for discrete variables.

Integration of qualitative and quantitative data primarily focused on comparing participants’ mental health need, based on quantitative scores on mental health and HRQoL measures, with their qualitative descriptions of their mental health status. Participants’ level of mental health need (high, moderate, low or none) was determined by meeting cut-off criteria for anxiety, depression, and/or PTSD symptoms, and whether their HRQoL score (MCS-8) was above or below population norms. This approach is based on previous evidence-based frameworks of health-seeking and mental health service use, that identify mental health need as a combination of both mental health symptom severity and quality of life, including perceived health and functional status [25]. Additional integration was used to triangulate findings on related constructs and is incorporated narratively within qualitative themes. Survey data for each participant was imported into NVivo, matched with their interview, and classified as attributes to explore relationships between qualitative codes and themes and the quantitative participant attributes [57, 58]. A detailed description of the integrated analysis procedures and joint display tables with findings are available in Online Resource 1. Findings are presented narratively in the results.

## Results

### Participant Characteristics

Participant characteristics including sociodemographic factors and criminal legal system involvement are provided in Supplemental Tables 1 and 2 in Online Resource 2. The average PHQ-2 score was 1.6 and 8 participants (27.6%) met criteria for depression. The average GAD-2 score was 1.5 and 7 participants (24.1%) met criteria for anxiety. The average PC-PTSD score was 2.4 and 15 participants (51.7%) met criteria for probable PTSD. The average HRQOL mental component score (MCS) was 45.3 (range 18.1–62.2) and 66% scored below 50 indicating poor HRQoL. Ten participants (34.5%) met criteria for at least 2 mental health conditions and had a low MCS-8 score which, taken together, indicated high mental health needs.

### Incarceration on Health-Seeking Behaviors

Five themes emerged describing how incarceration impacts participants’ health-seeking behaviors, recognition of mental health needs, coping behaviors, and engagement in formal resources in the criminal legal and community health care systems. These themes include (1) “I know what I need” focused on self-identification of participants’ mental health needs, (2) “You learn to depend on yourself” focused on use of individual coping behaviors to address mental health needs, (3) “Trying to express myself” focused on interactions with people in their social support network, including family and friends, (4) “How can you put your trust in that?” describes perceptions on seeking resources within the criminal legal system, and (5) “It’s not normal for guys like us” about experiences navigating healthcare resources post-incarceration. Findings of integrated data analysis are described narratively throughout the results.

#### Identifying Mental Health Needs: “I know what I need”

Overall, participants believed in their ability to identify their own mental health needs; this was reinforced by negative and discordant experiences with the outcomes of mental health evaluations before or during incarceration. Nearly all participants described receiving a mental health evaluation, most often as a part of a correctional facility’s intake process or while in solitary confinement. When asked about the outcomes or follow-up of the evaluation, few received any diagnoses, treatment, or support. Most stated that mental health evaluations solely focused on suicidal ideation, felt that these assessments were a formality to “check the boxes,” and conveyed a sense that no one really cared. Malcolm noted, “They’d listen. They’d ask the pertinent questions. Then they’d talk down to you. And then they forget all about you.”

Participants who received a mental health diagnosis during incarceration often did not understand or agree with the diagnosis. Describing his experience, John said, “The judge ordered me to have an evaluation. That was the evaluation. PTSD… I didn’t take it serious. I didn’t believe it. I didn’t start understanding mental health and believing it until I was locked up for a long period of time. I started reading up on it and studying it.” Similarly, Max stated, “I’m still trying to figure out why they had diagnosed me as being depressed. I didn’t feel depressed.” Whether they received a diagnosis or believed in the validity of diagnoses overall, participants often emphasized that they were the best arbiters of their mental health needs. Shawn stated:

At the end of the day, I know what I need. People can assess me, interview me, incarcerate me, observe me, and they can think they know what I need. They can say, ‘well, you know what, looking at him, based on the numbers he needs this.’ And that can be an educated assessment, but at the end of the day, I live inside of this body, inside of this head. I know what I need.

The way participants described their current mental health status provided insight into how they evaluated their own mental health needs. Often their response followed a distinct pattern: starting with an overall status such as “I’m stable,” or “I’m at peace,” acknowledging difficult circumstances including financial struggles or loss, and then concluding with coping strategies, resources, or other positive factors that provided balance amid adversity. Kenny exemplified this pattern, saying:

I’m doing good. I wanna say blessed. I have my rough times. That’s more so family issues. I’m trying to get a business off the ground, financially. I will say that it’s a struggle, but it’s not to the point where I’m desiring to rob, kill, steal for money – I actually do know where my next meal is. I have a roof over my head, a family. So I’m grateful for that. So, in the midst of my mother just passing away, my aunt in the hospital right now, still battling the death of my father a few years ago, I’m blessed.

Even among men who described very distressing situations, like witnessing a recent shooting or feeling overwhelmed, they still followed these descriptions with “I’m alright.”

Participants shared the particular distress associated with transitioning back into the community after incarceration and described moments where they identified mental health needs that led to them seeking formal mental health treatment. Antonio described, “Just felt like walls was closing in on me. Nobody truly understands.” Duss explained:

I was distraught. I was so down and out and low. I had attempted to cut my wrists and everything. But I walked myself into the hospital because in the breaking moment time, I realized I can’t fight this right now.

Participants who had not previously engaged in mental health treatment described indicators for when they would seek formal mental health treatment, such as harming themselves, lashing out at others, “living on the street,” or not being able to take care of their needs. Some explained they would simply “just know.” Rob stated, “I would know, just like knowing you gotta go to the bathroom, I’d know when something wrong. I can tell when something not right with me.”

The nuances of participants’ approach to self-assessment was also evident when comparing participants’ qualitative descriptions with their quantitative scores on mental health and HRQoL measures (see joint display table in Online Resource 1). Many participants categorized as having high mental health needs based on mental health measures, qualitatively described their mental health in positive or neutral terms. For instance, Antonio, who met criteria for depression, anxiety, and PTSD and had an MCS-8 score of 18 (indicating poor HRQoL) stated, “I’m pretty good as far as my mental health.” He acknowledged some experiences with loss and illness in his family and continued, “but I’m pretty stable.” Conversely, others whose self-assessments aligned with their measured needs were more direct about their struggles. Mike, whose MCS-8 score of 31 and positive screenings for PTSD, depression, and anxiety suggested high mental health needs, stated, “I know I got some issues with anger, patience. I got some issues with trusting human beings. In there, I got a lot of trauma from being in there.” Overall, these findings underscore dissonance and inconsistency between participants’ mental health scores and their self-perceptions.

#### Individual Coping Behaviors: “You learn to depend on yourself”

The theme, “you learn to depend on yourself,” describes individual behaviors deployed to cope with negative emotions or circumstances. For most, incarceration reinforced the need for self-reliance in addressing mental health needs due to limited agency and supportive connections. Most participants shared sentiments similar to Ken’s statement, “When you’re in prison you learn to depend on yourself.” Self-isolation was common, often discussed as a strategy participants picked up while incarcerated. Anthony shared, “jail had me like isolated, that’s what it did to me.” Participants described isolating themselves to avoid or escape distressing people or situations, or to actively problem solve for the things within their control. Malcolm shared his experience while living in a shelter post-incarceration, “I was up and out of there every day at 9:00 pounding the pavement, trying to improve my situation. This is every day. Even when I was incarcerated, I was very studious. I was always in my books. I was always trying to improve my situation.”

Many participants coped by staying busy or distracting themselves through work. Carl illustrated this, saying, “I’ll paint a whole house in one day if they let me stay there overnight. I dive into my work, … That gets me through.” However, some participants shared instances when staying busy was not helpful and they had to shift their coping strategies. Duss, who discussed working extra shifts as a certified nursing assistant (CNA) shared:

Doing the CNA work, I was sitting there talking to people like I’m a counselor, but I take on the stress of other people … it can wear and tear on you. So sometimes I take some time off from work a lot because that puts me in a state of depression too.

Several participants described smoking cannabis (commonly referred to as “marijuana” or “weed”) to address their mental health needs. Carl stated, “I’m an avid marijuana smoker – it calms me down. If I’m agitated, I smoke me a blunt and I’ll start writing, and that takes me to another place.” Tyrese shared that weed helped alleviate his anxiety after experiencing seizures:

I had a seizure almost two/three months ago… I was anxious. My palms were sweaty. Every time I come home from the hospital, I don’t want to stay in the house by myself. When I smoked, I was actually fine.

Several participants discussed that smoking weed helped them to problem solve. Mark shared, “I be snapping but when I smoke, I think better after, and I slow down. I reevaluate things.” Thomas shared, “I have a marijuana license. I got it because it was a time I had depression. I don’t no more. It was a time that I had anxiety real bad. I do sometimes. I do. … I can do it in a legit way, it’s more relaxing. I ain’t got to sneak. It’s just medicine for me now.”

Incarceration introduced or reinforced specific coping behaviors or activities including exercising, praying, reading, journaling, and meditating. For many participants, these activities were existing hobbies and interests utilized to cope since their incarceration, or new activities they were exposed to while incarcerated and that they continued after release. Tay, who participated in a military-style bootcamp program while incarcerated explained:

I work out. Any time I need to think, I go for a run to just calm myself down … I always was into being active. I used to go bike riding and swimming. I was on the track team when I was younger. I was always into being active. I learned how to use that to cope with my emotions, working out at the bootcamp. I learned how to use that tool.

JD also shared, “lot of that came while I was incarcerated, as far as the faith-based support and recovery … It’s very spiritual behind the walls, though. Yeah, you learn a lot because you’ve got the time to come closer to your Lord.” For many participants, the mental health harms of being incarcerated forced them to recall, reinforce, or learn new individual behaviors to cope with their mental health needs.

#### Navigating Interpersonal Resources: “Trying to express myself”

The theme “Trying to express myself,” reflects participant’s health-seeking experiences at an interpersonal level. Many participants described talking to their social support network, mainly friends and family, in order to “do things differently.” They described facing challenges when navigating the right ways to express themselves and the right people to confide in.

Participants shared that incarceration highlighted the need for talking to others to avoid the consequences of hiding or bottling up emotions. While describing past health-seeking behaviors participants often said they “just reacted” and had “all or nothing thinking,” which contributed to impulsive or aggressive behaviors. Duss described how his behavior has changed:

I’m not violent no more, I don’t snap at people no more…I still got a temper. But I’ve learned to not act out. I use my words more than my physical being… I’ve learned to convert all that physical stuff into a mental state, [I’m] more about finding a positive solution than anything now.

Some men described the ways in which their past suppression of emotions prompted explosive emotional responses which contributed to their incarceration. They shared that expressing themselves was essential to avoid reincarceration. David explained:

Or you have people that – in my situation, they’ve let a lot of stuff build up and then they’ll go outside and then lash out on the first person you see, then now they look up, that person’s no longer here and now you’ve got to spend the rest of your life in jail over an accident or because you wasn’t able to be vocal and express yourself… I’m getting more comfortable with expressing myself, whether it’s to my mom or if it’s to a friend or something like that. I just try to find an open ear that I can get.

Connecting with other incarcerated individuals helped participants take accountability, learn from shared experiences, and work to improve themselves and one another. Thomas described a group therapy program that he was in while incarcerated, “You all learned from each other. You all called each other out on your behaviors with them, and that was intense.” Kenny also shared:

I got around a few guys that it wasn’t just we talked about the Bible in church. When we ran the track we talked about the Bible. When we ate food we talked about the Bible, we talked about our family, we talked about our situations, like that… I’m grateful to be out right now. It’s a few guys that I did some time with that we still discuss things, not only our life, but we encourage each other with scripture and we talk about our issues.

While some were able to maintain these relationships post-incarceration, others were barred from contacting currently incarcerated people or those with records due to parole/probation requirements—limiting sources of social support.

After incarceration, participants described mixed experiences talking to people in their social support networks. Some participants felt that no one understood or cared about what they were experiencing. Shakur shared, “I try to express myself every day. People laugh and make a joke out of it… If I had somebody sitting one-on-one, talking to me about my problems, I’d feel better. Because when I talk to my friends or anything, they don’t care.” In contrast, others found support among friends, family, or romantic partners, often seeking help after initially trying to cope independently. Tyrese explained, “I learned how to cope with stuff by myself. If I need to talk to somebody, I will go around the corner and talk to my cousin.”

Most men emphasized the importance of being selective and intentional with whom they talked. Shawn shared:

Luckily – not luckily – intentionally, I’ve built a network, a good support network that I can go to if I’m in mental trouble, and I have people that I can talk to and that’ll talk me down off the ledge. Nobody will let me jump because they know I’ve invested in myself.

Participants described their desire to speak to those with similar experiences because they were more likely to understand their struggles, however, this was not without challenges. Thomas, in reference to relationships with women in his community, explained, “When we come back to them, we come back to them broken. And they trying to fix us, but they don’t know how to fix us. They’re broken too.” Participants suggested that FIBM did not always respect each other’s advice and would be more receptive of advice coming from Black women than Black men. Reese stated, “When it comes to Black men talking to Black women, if you notice, we more open with Black women than anything. We look at y’all like the backbone of the Black race.” The experience of incarceration prompted participants to seek mental health support from family, friends, and peers. However, finding the right individuals to whom they could express themselves remained a challenge for many.

#### Seeking Treatment Within the Criminal Legal System: “How can you put your trust in that?”

The theme “How can you put your trust in that” highlights how incarceration experiences deepened participants’ mistrust of healthcare, both within and beyond correctional settings. This mistrust stemmed from personal experiences and reflections on historical injustices that underscored systemic exploitation and discrimination. Carl articulated this sentiment:

Being a Black man living to 62 years old, I don’t trust the government from the Tuskegee experiment to the thing they had going on in Holmesburg prison. Before they closed that down they was doing experiments on Black people … How can you put your trust in that?

Most participants expressed that their negative or cautious perspectives on mental health treatment stemmed from the perception that psychiatric medications were the only treatment available during incarceration. Frank, who spent 46 years incarcerated, shared “they don’t really help you much. Their assessment of things is to take a pill, a pill for this, a pill for that. And that’s it… That’s their whole thing, a pill.” Tony shared that he was diagnosed with depression and PTSD upon incarceration but stated:

I ain’t never went to receive no treatment because I always thought they’re gonna put you on a bunch of pills and psych meds and all that, I know that’s what they do. I think they’re harming people when they be doing that for real if you ask me.

Participants frequently described mental health care while incarcerated as obligatory or punitive. Several were enrolled in mandated treatment programs during and after incarceration. They shared that they were just “going through the motions” and believed staff providing those programs did not care about participants’ mental health or well-being. Mike shared, “it’s really like a formality. You don’t get the mental health help that you need. They give you the bare minimum. … It’s not there to rehabilitate you, none of that. It’s more focused on punishment now.” Participants described experiencing or witnessing mistreatment or punishment tied to seeking mental health treatment during incarceration. JD shared, “They put you in a room and they would leave you there until you act. ‘You want to kill yourself?

All right. Well, let’s strip you, place you here, feed you the medication and put you on watch. And we’ll see how you act.’” Anthony shared,

I just don’t like the outlook on mental health and the type stigma they put on you… What they call it in jail is, ‘they’ve coded.’ I done seen people get coded and they don’t let them outta jail because they coded. They went to seek help and now when they want to seek help, it’s like they getting punished for that because they don’t ever get to come home.

A few participants enrolled in mandated treatment noted positive aspects, including being able to connect with others in group programming and having some agency in the process. JD stated, “Although, I’m stipulated they’re just not forcing me.” Some participants enrolled in mandated treatment expressed that they would have sought treatment even if it were not mandated.

When offering suggestions for programs or resources to help FIBM improve their mental health, a few men suggested structured or mandated programs without the threat of punishment. Thomas stated, “I think there should be a mandate to say, listen, you got to complete a year of mental health. But it should be an opportunity behind it as well, not a fault. Not ‘oh, you going back to jail’ – no. It should be an opportunity… Make a beneficial stipulation. You already told what to do to a certain degree so why not throw something in that’s going to be beneficial to do.” Though few participants sought mental health resources through the criminal legal system, when asked how they would go about seeking mental health treatment of they needed it, one participant stated he would “ask the courts.”

#### Navigating Healthcare Resources: “It’s not normal for guys like us.”

The theme, “It’s not normal for guys like us” reflects participants’ perceptions that seeking formal mental health services was atypical for Black men. David shared, “it’s not normal for guys like us as far as being Black, African American, to reach out to a therapist and stuff like that when we feel low.” Mike echoed this sentiment and describing stigma and mistrust associated with seeking mental health services, “We’re looked upon as if we’re weak if we talk about our problems…that’s the biggest thing, that stigma and that mistrust that came from years of oppression and things we went through as Black people.”

Despite these perceptions, twenty participants (69%) reported receiving formal mental health services at some point in their lives, often framing it as a deviation from social norms. Several participants sought treatment due to significant mental health symptoms, including suicidality, or a desire to improve their relationships. Mike described seeking help “so I could be a better husband, I could be a better father to my children, and things like that, a better citizen. I would go. Just to better myself.”

Motivation to seek treatment did not consistently prompt timely engagement in mental health treatment. In surveys, more than a third (37.9%) of participants reported putting off, waiting, or postponing seeking mental health treatment that they felt they needed, and 27.6% said there was a time they needed treatment but did not get it. Barriers included social norms and stigma, distrust of the healthcare system including concerns about racism, capitalism, and “Big Pharma,” as well as logistical issues such as scheduling difficulties, long waits, and distance. Six participants (20.7%) reported that they had experienced race-based discrimination, and described receiving minimal care and attention, having their experiences being ignored, and even noting differences in care when they were in a hospital in a “white-people zone” compared to hospitals in predominately Black or socioeconomically disadvantaged areas. Seven participants (24.1%) reported experiencing discrimination in health care settings due to their criminal record, and they described receiving the “bare minimum” or being questioned or criticized for their incarceration history.

Factors that facilitated treatment engagement included having health insurance and being engaged with reentry and related social services. All participants had health insurance, largely through Medicaid, and most (*n* = 25, 86%) had a primary care provider though they reported varying levels of engagement in primary care. Several described being connected to health insurance via a case manager or social worker as a part of a reentry program. Men who voluntarily sought mental health treatment walked into a crisis response center (CRC) or local clinic, asked their friends, or “googled it.” David stated “I basically just used Google for the most part – looked up therapy, therapy places, therapists, and I found a few places, I called around. Some were booked up, and the first ones that took me, I just went.” Thomas shared, “I went through my healthcare. And they hooked me up with [local clinic]. And I got a therapist.” Proximity to and the visibility of specific mental health clinics emerged as common facilitators. Participants mentioned walking or driving by clinics that not only were conveniently located but also prominently advertised walk-in hours and other services. This regular exposure reinforced their awareness of local mental health resources. Race and/or gender concordance with a mental health provider was also a facilitator. Mike explained, “If I go see someone that I’m comfortable with. Another Black person. Maybe another man. I mean, they might not understand my struggles either, but I’ll feel more comfortable talking to someone that looks like me and may have gone through the same thing I went through.”

Participants described changing insurance access due to un- or under-employment and expensive copays as barriers after incarceration. Kenny described “I did [counseling] for eight or nine months. And then, what happened was my insurance changed. So, I stopped. And when I had the new insurance, because of the copays, I didn’t go back to it.” Kenny only returned to therapy a few years later after the death of his father and another insurance change. Participants discussed that barriers to mental health treatment, including stigma, mistrust, and logistical issues, persisted years after being released from incarceration. These challenges reinforced the perception that formal mental health care remained largely inaccessible, even for those with resources and a willingness to seek formal mental health treatment despite social norms and stigma.

## Discussion

Participants described complex ways in which incarceration influences health-seeking behaviors and mental health after release. Foremost, incarceration exacerbated mental health needs, aligning with prior research that characterizes incarceration as an inherently traumatic and mental health-harming experience that limits agency, increases social isolation, and contributes to additional violence exposures and socioeconomic disadvantages [1, 21, 38, 59–61]. Further, the distress of transitioning back into the community can be immensely overwhelming and contribute to mental health crises [21, 35, 59, 62].

One component of health-seeking is identifying mental health needs. Participants often rejected mental health evaluations conducted within the criminal legal system, perceiving them as inadequate or lacking justification. Aligning with previous research, participants’ experience of receiving unclear, inappropriate, or frequently changing diagnoses eroded trust in mental health services [33]. The integrated analysis highlighted incongruence between how FIBM assessed their own mental health and how it was measured through conventional screening tools. This complexity has been described in other studies [16, 36] and suggests that established biomedical approaches to assessing FIBM’s mental health warrant further consideration, especially in the context of structural racism [15, 18, 63] and sociocultural influences [23, 64, 65]. Moreover, the experience of incarceration reinforced participant’s reflexive self-reliance rather than outreach for identifying mental health needs and they were self-assured in their ability to respond accordingly.

For some participants and in some interpretations, incarceration promoted health-seeking behaviors by bolstering coping strategies and introducing new ones, as men realized that they had to depend on themselves. While faith-based programming in carceral settings and religious or spiritual coping has been documented in previous studies [66–68], participants in this study also discussed exercising, reading, and writing as coping behaviors they used during and after incarceration. Some of these coping behaviors stemmed from educational, vocational, and recreational programming in correctional facilities which, though not explicitly designed for mental health support, fostered well-being through skill-building and social connection. Participants described incarceration as a turning point, prompting them to reconsider past coping mechanisms and, in some cases, seek support from social networks or mental health services. These findings align with existing research on the importance of self-reliance, resilience, social networks, and additional sociocultural considerations in shaping Black men’s health-seeking behaviors, while highlighting how incarceration uniquely influences these dynamics [23, 65, 69].

The themes, “how can you put your trust in that” and “it’s not normal for guys like us,” outlined how participants navigated seeking formal mental health services, within both the criminal legal system and community-based health care system. Participants’ references to the Holmesburg Prison experiments, which took place at a Philadelphia prison in the mid-to-late 20^th^ century [70, 71], highlighted the significance of local and contemporary examples of race-based medical exploitation in shaping their perceptions of formal systems. These examples, compounded by personal experiences of mistreatment and discrimination, deepened participants’ mistrust and negative perceptions of formal mental health services. As others have argued, it is critical to go beyond widely cited cases like Tuskegee to examine how personal, localized, or institution-specific histories may inform engagement with criminal legal and health systems [72]. Findings indicated that despite widespread distrust and harmful experiences, the criminal legal system was still perceived by some FIBM as the primary or only pathway to mental health services which is underexplored in existing literature. Though many participants engaged with community-based mental health services due to parole or probation mandates, many also voluntarily sought treatment. While access to services was possible through insurance and knowledge of available resources, structural barriers such as underinsurance, high copays, long wait times, stigma, and discrimination based on race and criminal records persisted - consistent with prior research on barriers to care for FIBM [34–36, 59]. This study further highlights that these barriers extend beyond the immediate post-incarceration period, limiting access to quality mental health services even years after release.

### Limitations

Findings of this study should be interpreted within the context of its limitations. The use of convenience and snowball sampling methods resulted in participants within similar social networks and local reentry programs. This may have influenced their engagement with mental health services, potentially overrepresenting those inclined to seek support. However, given that participants were recruited from and embedded within structurally disadvantaged contexts similar to what has been described in other studies [35, 59, 62], their experiences accessing and engaging resources may provide insights into how to promote health-seeking behaviors among FIBM without comparable resources. Second, the categorization of participants into high, moderate, low, or no need groups for integrated data analysis was not based on previously validated procedures, and was instead informed by the research and clinical expertise of the coauthors. The categories align with existing literature that illuminates how individuals exist on a spectrum of mental health morbidity ranging from relatively “healthy” to high mental health morbidity with intermediate groups reflecting varying levels of mental health need [73, 74]. This approach facilitated meaningful, mixed methods integration of the findings.

### Implications and Conclusion

Efforts to improve mental health services in correctional facilities have emphasized the need for increased funding, training, and capacity to address the mental health needs of all incarcerated individuals, regardless of formal diagnoses or apparent symptoms [31, 33]. However, as reflected in both existing literature and participant experiences, the implementation of these policies remains inconsistent, often leading to inadequate or punitive responses to mental health needs [33]. Given participants’ experiences with mental health services, programs and services for currently and formerly incarcerated individuals, including those without a direct mental health focus, should be critically evaluated to ensure that they provide meaningful mental health promotion rather than reinforce harm or mistrust. Additionally, mandated or court-ordered mental health treatment should be examined through critical and anti-racist frameworks to minimize punitive approaches and ensure that care aligns with the needs of formerly incarcerated individuals and broader efforts to address mental health inequities [28, 75, 76].

This study identified facilitators of mental health care access with broader policy implications. Although all participants had health insurance, barriers such as underinsurance, high copays, and coverage gaps persisted. Continuous and comprehensive health insurance coverage for formerly incarcerated individuals is essential to reducing structural barriers to care [33, 77–80]. Findings underscored the importance of visibility in increasing awareness of local mental health services. Participants often named specific, recognizable clinics including those offering walk-in services, suggesting that these facilities’ physical presence and integration within the community enhanced the perceived accessibility of mental health treatment. Increasing service visibility, coupled with proactive outreach, could improve engagement and reduce stigma associated with seeking mental health care [27, 81]. Mental health providers and treatment settings should educate communities about available services, levels of care, and treatment goals to enhance mental health literacy and facilitate engagement in mental health services [80, 81].

Participants’ experiences of perceived racial discrimination and broader structural racism, at the intersection of the criminal legal and healthcare systems reinforced the need for anti-racist policies and practices across both sectors [29, 75]. Participants’ preference for race- and gender-concordant providers to foster trust and engagement aligns with prior research demonstrating improved trust and outcomes in racially concordant patient-provider relationships [82–85]. Beyond these concordant patient-provider relationships, cultural responsiveness and attention to sociocultural factors are necessary to ensure meaningful engagement [23, 65, 69]. Furthermore, integrating FIBM as peer support specialists or navigators within healthcare and reentry services may further enhance trust, engagement, and continuity of care by providing culturally responsive support from individuals with shared lived experiences [38, 86–88].

This study underscores the ways in which FIBM identified and responded to mental health needs through their individual coping strategies and engagement with social support and mental health treatment across correctional and community settings. Findings reinforce previous research that emphasizes the need for tailored interventions that address the uniquely disproportionate impact of incarceration for Black men in the U.S. By centering the lived experiences, agency, and internal resources of FIBM, interventions can be designed to strengthen coping skills, enhance social support, and improve access to care. Building on this work is essential to mitigating the mental health harms of incarceration, preventing downstream health consequences, and improving the overall well-being of FIBM.

## Supplementary Material

Supplementary 2

Supplementary 1

**Supplementary Information** The online version contains supplementary material available at https://doi.org/10.1007/s40615-025-02591-7.

## Data Availability

The data that support the findings of this study are not publicly available due to the sensitive and identifiable nature of the qualitative interviews, and in accordance with ethical approval and participant consent.
