# Supplementary material for: “I Know What I Need”: A Mixed Methods Study of Mental Health-Seeking Behaviors in Formerly Incarcerated Black Men
Source: J Racial Ethn Health Disparities. 2025 Aug 13;13(6):4422–34. doi: 10.1007/s40615-025-02591-7 (PMC13292832; doi:10.1007/s40615-025-02591-7)
Supplement: Supplementary file 1 [file 40615_2025_2591_MOESM1_ESM.docx]

Manuscript Title: “I Know What I Need”: A Mixed-Methods Study of Mental Health-Seeking Behaviors in Formerly Incarcerated Black Men

Journal: Journal of Racial and Ethnic Health Disparities

This document, Online Resource 1, provides a detailed overview of the integrated data analysis procedures, along with a joint display table illustrating the results. Findings were reported and interpreted narratively in manuscript.

**Integrated data analysis procedures**

| Goal of analysis:  How did participants’ scores on mental health symptom severity measures and HRQoL compare to their qualitative descriptions of their mental health status? | | |
| --- | --- | --- |
|  | Quantitative | Quantitative |
| Data collection | Open-ended Interview Questions:   - In general, how are you doing? - What does mental health mean to you? - How would you describe your own mental health right now? - How is your mental health affecting other aspects of your life? | Mental health measures   - PHQ-2 (Depression symptoms) - GAD-2 (Anxiety symptoms) - PC-PTSD-5 (PTSD symptoms) - MOS SF-8 (Health-related Quality of Life) |
| Data analysis | Using NVivo,   1. Qualitative data was initially analyzed using open, inductive coding. 2. Participants’ qualitative descriptions of their current mental health were coded into three codes: "generally negative," "neutral/okay," and "generally positive." | Using Qualtrics and Stata,   1. Quantitative scores were calculated for each mental health measure. 2. Scores were dichotomized based on cut-off values for probable depression, anxiety, or PTSD. HRQoL MCS-8 scores were dichotomized in relation to population norms (above/below 50). 3. Participants were categorized into **High**, **Moderate**, **Low**, or **No Needs** based on predefined criteria:    a) High: MCS-8 score below 50 AND meets criteria for 2 or more mental health conditions.    b) Moderate: MCS-8 score below 50 AND meets criteria for 1 mental health condition    c) Low: MCS-8 score below 50 OR meets criteria for 1 mental health condition    d) No/none: MCS-8 score about 50 AND does not meet cut-off criteria for further screening for anxiety, depression, or PTSD. |
| Data management |  | Participants’ scores on each measure and need category were imported into NVivo attributes and matched with participant interviews [1,2]. |
| Integrated Analysis   1) Cross-tabulated mental health need categories (High, Moderate, Low, None) with qualitative sub-codes (generally negative, neutral, positive). 2) For each need category, qualitative sub-codes and extracted quotes were examined to identify areas of congruence or incongruence. For example, congruence meant participants with high mental health symptom severity scores also had qualitative descriptions of mental health status were coded as “generally negative.” Incongruence referred to those with high mental health symptom severity whose qualitative descriptions of their mental health status were “generally positive.” | | |

**References**

1. Andrew S, Salamonson Y, Halcomb EJ. Integrating mixed methods data analysis using NVivo: An example examining attrition and persistence of nursing students. Int J Mult Res Approaches. 2008;2(1):36–43. https://doi.org/10.5172/MRA.455.2.1.36

2. Swygart-Hobaugh M. Bringing method to the madness: An example of integrating social science qualitative research methods into NVivo data analysis software training. IASSIST Q. 2019;43(2):1–16. https://doi.org/10.29173/iq956

**Joint Display Table – Mental health status (QUAL) by mental health needs (quant)**

|  | Participants’ own descriptions of mental health status | | |
| --- | --- | --- | --- |
|  | **Generally Negative**  (n=3)  Participant’s own description of mental health status coded as generally negative. | **Neutral/Okay** (n=9)  Participant’s own description of mental health status coded as neutral or okay. | **Generally positive**  (n=17)  Participant’s own description of mental health status coded as generally positive. |
| High Need:  (n=10) | Congruent  2 participants | Incongruent  5 participants | Incongruent  3 participants |
| Moderate need:  (n=5) | Congruent  0 participants | Congruent  2 participants | Incongruent  3 participants |
| Low need:  (n=6) | Incongruent  1 participant | Congruent  1 participant | Congruent  4 participants |
| Negligible/No Need:  (n=8) | Incongruent  0 participants | Congruent  1 participant | Congruent  7 participants |
