# Supplementary material for: “I Know What I Need”: A Mixed Methods Study of Mental Health-Seeking Behaviors in Formerly Incarcerated Black Men
Source: J Racial Ethn Health Disparities. Author manuscript; Available in PMC 2026 Jun 24. (PMC13292832; doi:10.1007/s40615-025-02591-7)
Supplement: Supplementary 2 [file NIHMS2188134-supplement-Supplementary_2.docx]

Manuscript Title: “I Know What I Need”: A Mixed-Methods Study of Mental Health-Seeking Behaviors in Formerly Incarcerated Black Men

Journal: Journal of Racial and Ethnic Health Disparities

This document, Online Resource 2, provides detailed tables describing the quantitative findings of this study, including sociodemographic characteristics, criminal legal involvement, mental health measures, and additional survey questions.

**Supplemental Table 1. Sociodemographic Characteristics**

|  |  |  | n | (%) |
| --- | --- | --- | --- | --- |
| Age | |  |  |  |
|  | 18-24 |  | 1 | 3.4 |
|  | 25-39 |  | 13 | 44.8 |
|  | 40-54 |  | 7 | 24.1 |
|  | 55+ |  | 8 | 27.6 |
| Education | |  |  |  |
|  | Less than HS diploma |  | 6 | 20.7 |
|  | HS diploma or GED |  | 12 | 41.4 |
|  | Some College or College Degree |  | 9 | 31.0 |
|  | Vocational Degree or certificate |  | 2 | 6.9 |
| Employment | |  |  |  |
|  | Unemployed and looking for work |  | 8 | 27.6 |
|  | Unable to work/Disability |  | 6 | 20.7 |
|  | Employed part-time |  | 6 | 20.7 |
|  | Self-employed |  | 5 | 17.2 |
|  | Employed full-time |  | 3 | 10.3 |
|  | Retired |  | 1 | 3.4 |
| Health Insurance | | | | |
|  | Medicaid |  | 28 | 96.6 |
|  | Medicare |  | 3 | 10.3 |
|  | VA-Tricare |  | 1 | 3.4 |
|  | No health insurance |  | 0 | 0 |
| Primary Care Provider | |  |  |  |
|  | Yes |  | 25 | 86.2 |
|  | No/Unsure |  | 4 | 13.8 |
| Notes: Health Insurance is greater than 100% due to some participants having two types of health insurance. | | | | |

**Supplemental Table 2. Criminal Legal System Involvement**

|  |  |  | n | (%) |
| --- | --- | --- | --- | --- |
| Age of first incarceration | |  |  |  |
|  | ≤ 17 |  | 14 | 48.3 |
|  | 18-24 |  | 12 | 41.3 |
|  | 25+ |  | 3 | 10.3 |
| Total number of times incarcerated | |  |  |  |
|  | Once |  | 7 | 24.1 |
|  | 2-5 times |  | 10 | 34.5 |
|  | 5 or more times |  | 12 | 41.3 |
| Total amount of time spent incarcerated | |  |  |  |
|  | Less than 1 year |  | 5 | 17.2 |
|  | 1-5 years |  | 6 | 20.7 |
|  | 5-10 years |  | 4 | 13.8 |
|  | 10-20 years |  | 9 | 31 |
|  | 20 years+ |  | 5 | 17.2 |
| Times since last incarceration | |  |  |  |
|  | Less than 12 months |  | 11 | 37.9 |
|  | 1 year to 5 years |  | 9 | 31 |
|  | More than 5 years |  | 9 | 31 |
| Current Community Supervision Status | |  |  |  |
|  | Parole |  | 8 | 27.6 |
|  | Probation |  | 8 | 27.6 |
|  | Other |  | 3 | 10.3 |
|  | None |  | 10 | 34.5 |
| Notes: “Other” for community supervision includes house arrest, and compassionate (medical) release. | | | | |

**Supplemental Table 3. Survey Findings**

|  | | n (%) or Mean ± SD |
| --- | --- | --- |
| Mental Health Measures | | |
|  | Depression Score |  |
|  | Mean ± SD | 1.55 ± 1.66 |
|  | Depression Score ≥ 3 | 8 (27.6) |
|  | Anxiety Score |  |
|  | Mean ± SD | 1.52 ± 1.45 |
|  | Anxiety Score ≥ 3 | 7 (24.1) |
|  | PTSD Score |  |
|  | Mean ± SD | 2.41 ± 1.55 |
|  | PTSD Score ≥ 3 | 15 (51.7) |
|  | MCS-8 Score |  |
|  | Mean ± SD | 45.29 ± 10.36 |
|  | MCS-8 ≤50 | 19 (65.5) |
| Survey Questions | | |
|  | Have you ever felt discriminated against in healthcare settings because of your race? |  |
|  | Yes | 6 (20.7) |
|  | No | 23 (79.3) |
|  | Have you ever felt discriminated against in healthcare settings because of your criminal record? |  |
|  | Yes | 7 (24.1) |
|  | No | 22 (75.9) |
|  | Was there ever a time that you needed mental health care but did not get it? |  |
|  | Yes | 8 (27.6), |
|  | No | 21 (72.4) |
|  | Have you ever put off, waited, or postponed seeking mental health care that you needed? |  |
|  | Yes | 11 (37.9) |
|  | No | 18 (62.1) |
|  | Have you ever received any mental health treatment at any point in your life? |  |
|  | Yes | 20 (69.0) |
|  | No | 9 (31.0) |
